# Insights into the role of adipose-derived stem cells and secretome: potential biology and clinical applications in hypertrophic scarring

**DOI:** 10.1186/s13287-024-03749-6

**Published:** 2024-05-12

**Authors:** Menglin Wang, Jianyu Zhao, Jiacheng Li, Meng Meng, Mengru Zhu

**Affiliations:** 1 Department of Plastic Surgery, The First Affiliated Hospital, Dalian Medical University, No. 222, Zhongshan Road, Xigang District, Dalian, 116011 China; 2 Department of Plastic Surgery, The Second Affiliated Hospital, Dalian Medical University, Dalian, China; 3 Department of Orthopaedics, The First Affiliated Hospital, Dalian Medical University, No. 222, Zhongshan Road, Xigang District, Dalian, 116011 China

**Keywords:** Hypertrophic scars, Adipose-derived stem cell, Stem cell therapy, Mechanism

## Abstract

Scar tissue is the inevitable result of repairing human skin after it has been subjected to external destructive stimuli. It leads to localized damage to the appearance of the skin, accompanied by symptoms such as itching and pain, which reduces the quality of life of the patient and causes serious medical burdens. With the continuous development of economy and society, there is an increasing demand for beauty. People are looking forward to a safer and more effective method to eliminate pathological scarring. In recent years, adipose-derived stem cells (ADSCs) have received increasing attention from researchers. It can effectively improve pathological scarring by mediating inflammation, regulating fibroblast proliferation and activation, and vascular reconstruction. This review focuses on the pathophysiological mechanisms of hypertrophic scarring, summarizing the therapeutic effects of in vitro, in vivo, and clinical studies on the therapeutic effects of ADSCs in the field of hypertrophic scarring prevention and treatment, the latest application techniques, such as cell-free therapies utilizing ADSCs, and discussing the advantages and limitations of ADSCs. Through this review, we hope to further understand the characterization of ADSC and clarify the effectiveness of its application in hypertrophic scarring treatment, so as to provide clinical guidance.

**Supplementary Information:**

The online version contains supplementary material available at 10.1186/s13287-024-03749-6.

## Background

Scar tissue is an inevitable result of human skin repair after the exposure to destructive external stimuli, and is related to race, gender, age, and the tension, location, and pattern of injury of the wound [1]. If the injury reaches deeply into the reticular layer of the dermis, an abnormal fibroproliferative response occurs leading to hypertrophic scarring (HTS), which may even go on to develop into a keloid [2]. The characteristics of HTS are usually associated with collagen overproduction and an altered ratio of type I collagen to type III collagen [3, 4]. Approximately 35% of patients with postoperative scarring are estimated to develop HTS within one year [5]. Scarring not only leads to localized damage to the appearance of the skin, but also often accompanied by symptoms such as itching and pain [6]. Severe scar contracture located in the functional area may also cause mobility problems for the patient, which greatly reduces the quality of life and creates a burden on life and psychology [7].

The pathogenesis of HTS has not yet been clarified, and it is generally accepted that it may be related to the persistent inflammatory response and abnormal proliferation of fibroblasts, excessive vascular regeneration, and excessive extracellular matrix (ECM) deposition [3, 8]. HTS usually occurs in areas where the skin has been stretched, protrudes from the wound site, and grows rapidly over a period of 4–12 weeks, and once formed is difficult to recover as before [9, 10]. Clinical treatment of pathologic scarring is complex and difficult, and currently available treatments include localized compression, laser therapy, steroid injections, and surgical excision [11]. However, these methods cannot completely avoid excessive scar tissue formation, nor can they regenerate healthy dermal tissue, and may lead to skin ulcers, pain, localized tissue atrophy, hyperpigmentation or hypopigmentation, and many other complications [5, 12]. With the continuous development of economy and society, people’s demand for beauty is increasing, and they are looking forward to a safe and effective way to combat pathological scarring.

Adipose-derived stem cells (ADSCs) are a type of mesenchymal stem cells (MSCs) present in adipose tissue, and their function has received increasing attention from researchers in recent years due to their multi-differentiation potential, which allows them to differentiate into adipose and osteoblastic tissues [13, 14]. When skin trauma occurs, ADSCs and mature adipocytes mediate the inflammatory response and regulate the proliferation and activation of fibroblasts through paracrine secretion of a variety of cytokines, which has a certain therapeutic effect on dermal fibrosis and refractory scarring [15–17]. In addition, progressive reduction of intradermal adipocytes is a common pathology in many dermatofibrotic diseases [9, 18]. Last but not least, most patients would welcome the removal of some excess fat [19]. These findings suggest that ADSCs may play an important role in dermal fibrosis and scar formation, and therefore it is important to gain insight into the exact nature of the therapeutic effects of ADSCs on scarring. This article focuses on the pathophysiological mechanisms of skin scarring, summarizes the therapeutic effects of in vitro, in vivo, and clinical studies of ADSCs in the field of skin scarring prevention and treatment, the latest application techniques, and discusses the advantages and limitations of ADSCs with the aim of guiding the clinic. For this purpose, we searched PubMed, Web of Science, and EMBASE using “adipose-derived stem cells” or “ADSCs” as well as three related keywords. We primarily searched for original research published in the last 10 years on cellular studies, animal studies, and clinical trials on hypertrophic scarring.

### Characterization of ADSCs

MSCs can be obtained from a variety of sites, including bone marrow, umbilical cord, and adipose tissue, but adipose tissue has emerged as the best source for harvesting MSCs [14]. Enzymatic digestion of adipose tissue produces many cells, including adipose-derived stem cells, preadipocytes, vascular endothelial cells, and fibroblasts, and this mixed cell population is known as stromal vascular fraction (SVF) [20]. ADSC was isolated from primary adipocytes by performing a passaging culture. In 2001, Zuk et al. first isolated ADSCs from the SVF of adipose tissue [21]. As a type of MSCs, ADSCs are more clinically attractive because they are easy to extract, less invasive, easy to obtain in large quantities, non-immunogenic, and do not involve ethical issues [14, 22]. It should be noted that ADSC has similar characteristics to bone marrow mesenchymal stem cells (BMSCs), but ADSC has a longer lifespan, higher proliferative capacity, shorter doubling time, and later in vitro senescence, which may be beneficial in the treatment of chronic or intractable diseases [23].

### Sources

**Fig. 1:**
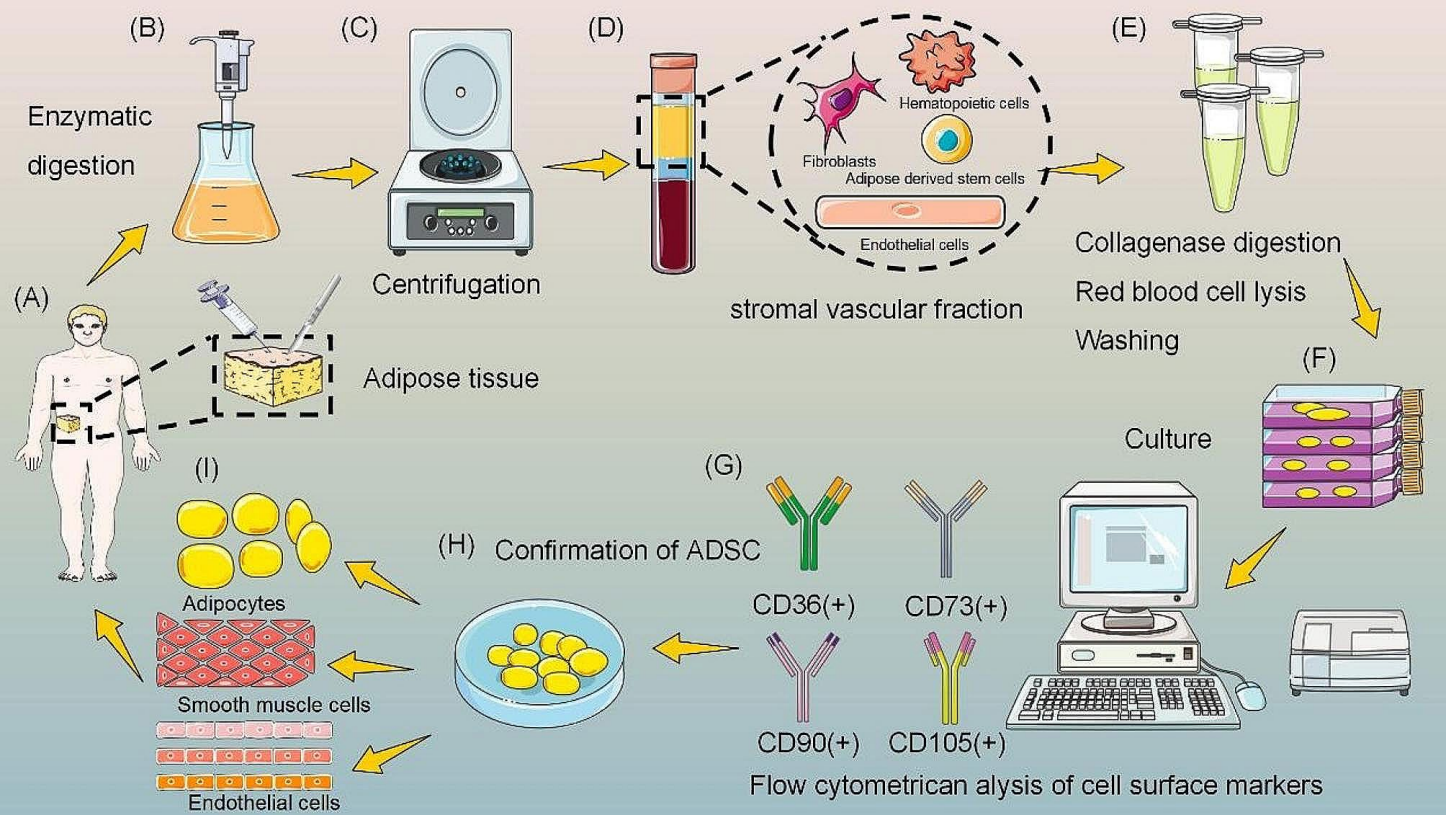
The process of harvesting, isolation, and characterization of ADSC. Adipose tissue was obtained by liposuction or surgical excision, digested by enzymes, and centrifuged to isolate the stromal vascular fraction. After collagenase digestion, red blood cell lysis, and washing, the stromal vascular fraction was cultured and analyzed by flow cytometry for the presence of cell surface markers to confirm the presence of ADSC characteristics. After culture, they are applied to the human body. Figure was drawn by the author

The abundant source of ADSCs is an important foundation for scar treatment research and application. First, with the improvement of living conditions, the rate of overweight and obesity has gradually increased, and liposuction and surgical removal of excess adipose tissue have made ADSCs more readily available [24]. Current methods of collecting ADSC include suction, mechanically assisted liposuction, power-assisted liposuction, laser-assisted liposuction, ultrasound-assisted liposuction, and surgical excision [14, 25]. Cells isolated by power-assisted techniques are characterized by high value-added potential and low senescence, and are therefore identified as the optimal approach [26]. Second, the isolation of ADSCs is relatively simple. In 1964, Rodbell described a method for isolating adipocytes from adipose tissue using collagenase, which became the “gold standard” [27]. Although trypsin, clostridial protease, and dispase are also used to isolate ADSCs, they may alter cell viability and reduce the regenerative potential of ADSCs [28]. Therefore, some scholars have attempted to use mechanical dissociation using scalpels or scissors, but the cell yield is low [29]. Finally, ADSC can proliferate stably in vitro, is easy to culture, and has a low mortality rate [29]. The whole extraction and culture process is shown in Fig. 1.

There are various types of adipose tissue in the human body, and ADSCs from different sexes and extraction sites have different proliferative, differentiation, paracrine, and anti-apoptotic capacities [30]. Subcutaneous adipose tissue is most commonly used for ADSC isolation because it can be obtained from the abdomen, thighs, and arms in a simple and non-invasive procedure [31]. Although it can also be extracted from areas such as the intrathoracic cavity, viscera, and retroperitoneum, studies have shown that ADSC from subcutaneous adipose tissue has a higher proliferative capacity and better anti-inflammatory effect [32]. In addition, tummy tucks are a popular site choice for surgeons because of their accessibility, abundance, and aesthetic improvement for patients. White adipose tissue is the source of ADSCs in most studies [29]. Brown adipose tissue has been thought to exist only in infants and to decrease with age [33]. However, recent studies have shown the presence of functional brown adipose tissue in adults with ADSC [32].

The age, weight, and disease state of the donor may affect the condition and nature of isolated ADSCs [34]. A study by Faustini et al. [35] showed that female adipose tissue obtained significantly higher ADSC production than male adipose tissue. In addition, ADSC obtained from obese donors showed increased proliferative and migratory capacity, excessive immune response, and reduced differentiation potential [36]. With aging, the proliferative and differentiation potential of acquired ADSCs is all over the place, and growth factor secretion becomes weaker [37, 38]. In addition, ADSCs provided by patients with diabetes, hypercholesterolemia, hypertension, and smoking were less pluripotent and self-renewing [31]. Platelet-rich plasma has been shown to improve human ADSC proliferation [39]. We believe it is necessary to use ADSCs isolated from healthy individuals to avoid poor efficacy and potential side effects.

### Phenotypes

In recent years, scholars have been working to discover ADSCs-specific surface antigens [29]. In 2013, the International Society for Cell & Gene Therapy (ISCT) and the International Federation for Adipose Therapeutics and Science (IFATS) established that the minimum criteria to define ADSC must express CD73(+), CD90(+), CD105(+), and CD36(+) [40]. The expression of CD36 and lack of expression of CD106 distinguish ADSCs from BMSCs. Notably, the phenotype of ADSCs in culture is dynamic. For example, CD34 has been shown to be at its highest level in early ADSCs, while its expression decreases throughout the culture period [41]. There is some controversy about CD34, whose expression on ADSC is unstable [42–44]. Some authors have confirmed that cultured ADSC does not express CD34 [45], while others have reported that some fractions of ADSC are CD34(+) [44]. Comparison of sorted fractions from early passages of cultured human ADSC showed that CD34(+) cells have a greater proliferative potential and colony-forming capacity, whereas CD34(-) cells are characterized by a greater potential to differentiate into osteoblasts and adipocytes [46].

### Adipose cell-free derivatives

ADSC exerts its therapeutic effects not only through direct cell-to-cell interactions, but also through the secretion of many molecules responsible for cell signaling, such as cytokines, growth factors, chemokines, extracellular vesicles, and other active substances [34, 47]. Cell-free therapies utilizing ADSC avoid the disadvantages of whole-cell drug delivery, such as potential tumorigenicity and storage issues [48]. Considering the effectiveness, safety, and cost aspects of ADSC, the dose and frequency of cell application cannot be increased indefinitely, therefore, ADSC-conditioned medium (ADSC-CM), ADSC-exosomes (ADSC-Exo), and other adipose cell-free rows of organisms have entered into the field of vision of researchers [49–52].

ADSC-CM is a wide-spectrum bioactive factor released by ADSC into the culture medium during cell culture and has been shown to promote wound healing and immunomodulation [53, 54]. ADSC-CM can reduce the cost of treatment and avoid the safety issues associated with stem cell therapy, but the short lifespan of the active ingredient, which is rapidly diluted and eliminated by diffusion, limits its use [55–57]. It has been reported that under hypoxic conditions, ADSCs may activate hypoxia inducible factor 1α (HIF-1α), thereby stimulating the proliferation of ADSCs and promoting the secretion of vascular endothelial growth factor (VEGF), hepatocyte growth factor (HGF) and basic fibroblast growth factor (bFGF) [58, 59]. In addition, ADSC-CM obtained using three-dimensional culture better mimicked the in vivo ADSC environment, containing more transforming growth factor-β1 (TGF-β1) and VEGF. Thus, hypoxia combined with three-dimensional culture could provide optimal culture conditions for obtaining ADSC-CM.

ADSC-Exos are small extracellular vesicles with a diameter of 30–150 nm [20]. As one of the components of paracrine signaling, they have a variety of activities that can penetrate physiological tissue barriers and participate in the exchange of substances and information between cells [60]. ADSC-Exo is enriched with proteins, lipids, and nucleic acids, which are involved in cell proliferation, apoptosis, immunomodulation, and remodeling of the ECM [61]. In addition, they are not rejected by the immune system and have a homing dose that is easy to control [55]. In contrast to ADSCs, ADSC-Exo is a novel cell-free therapeutic technique that circumvents the challenges and dangers associated with the use of natural or synthetic stem cells [60]. We speculate that paracrine signaling and direct cell-cell interactions may elicit different or even opposite responses in fibroblasts, which may partially explain why ADSCs themselves exhibit anti-fibrosis [12].

### Mechanisms of scar formation

**Fig. 2:**
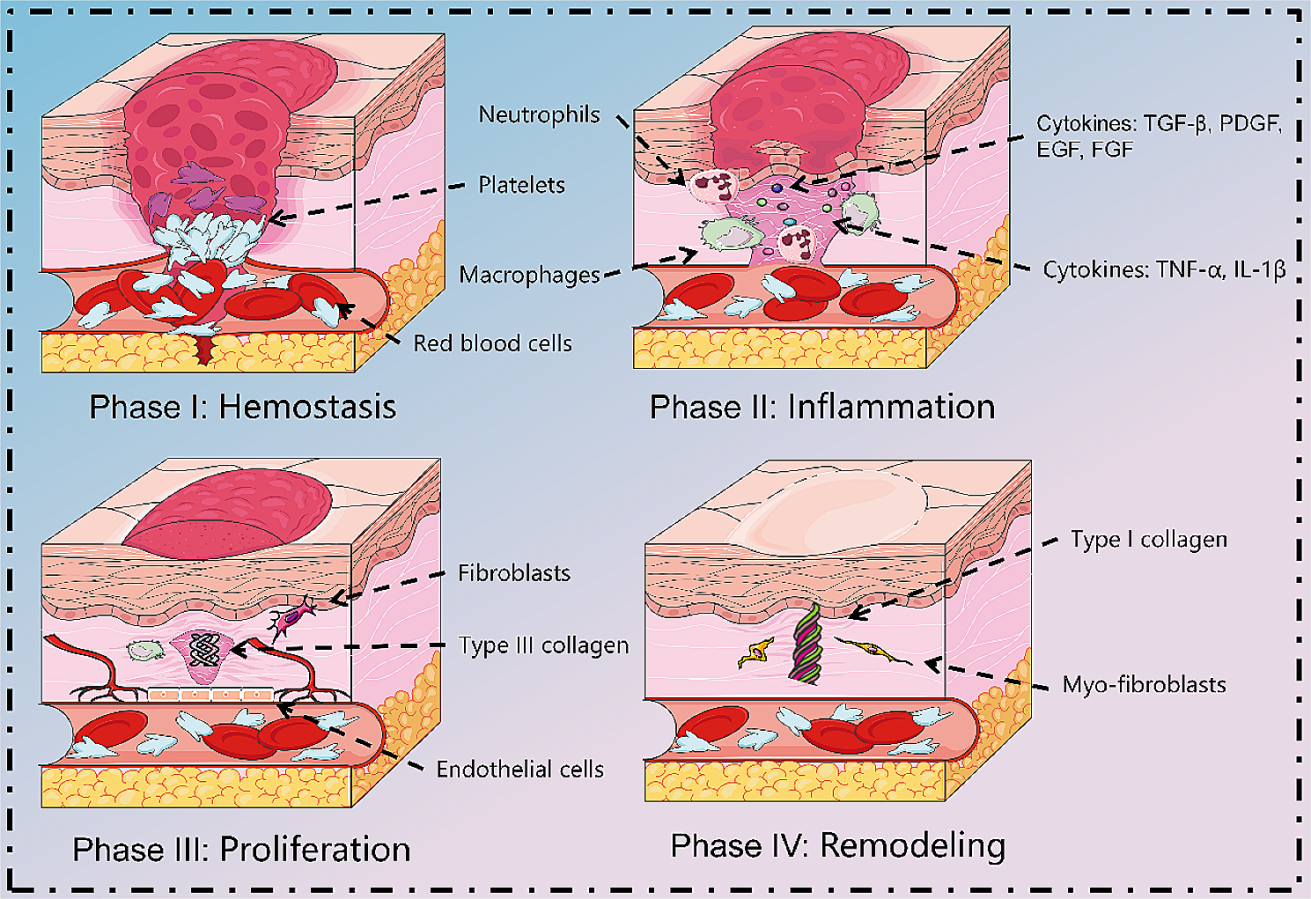
Normal healing process. Normal tissue repair involves many overlapping stages. After injury, hemostasis occurs initially through vasoconstriction and platelet aggregation. Subsequently, macrophages express inflammatory cytokines and chemokines, including tumor necrosis factor (TNF)-α, interleukin (IL)-1β, and IL-12, which recruit neutrophils and generate inflammation. Next, during the proliferative phase, macrophages promote tissue regeneration and ECM production by regulating the proliferation and migration of fibroblasts, and endothelial cells. As the tissue matures, neovascularization degenerates, and the ECM is rebuilt in the final remodeling phase. As granulation tissue is generated, fibroblasts differentiate into Myo-fibroblasts, which produce denser type I collagen and are responsible for wound contraction. Figure was drawn by the author

The wound healing process is divided into four successive and overlapping phases of hemostasis, inflammation, proliferation, and remodeling, involving interactions between cells and inflammatory mediators [16], as shown in Fig. 2. The normal wound healing process is transient, with most healing taking no more than 2 to 3 weeks. However, certain specific types of wounds, such as deep burns and infected wounds, form HTS characterized by extensive fibrosis due to prolonged healing time or the presence of a prolonged inflammatory response [18]. In molecular level, HTS has a large number of pro-fibrotic cytokines, inflammatory factors, etc. In histological level, it mainly consists of myofibroblasts (Myo-Fb) expressing α-smooth muscle actin (α-SMA) and ECM with predominantly type I collagen. It has a large number of inflammatory cells, immune cells, epidermal thickening with papilla reduction, and lacks normal skin appendages such as hair follicles, sweat glands, and sebaceous glands [62]. Changes in external tension and intracellular mechanical properties are associated with collagen, and scars are the specific result of collagen overproduction [63].

### Inflammation

Inflammation consists of a vascular response (hemostasis) and a cellular response (inflammation) that lasts for the first 4 days after injury. After skin breakage, hemostasis is achieved by vasoconstriction, platelet aggregation, and activation of the coagulation cascade reaction to form a fibrin clot, preventing the loss of pathogens and body fluids [64]. Fibrin clots and damaged tissues release cytokines (TGF-β, platelet-derived growth factor (PDGF), epidermal growth factor (EGF), FGF, and IL-8) to recruit neutrophils into the wound within 24–36 h [65]. Subsequently, neutrophils remove microbial pathogens by secreting proteases to digest damaged tissue [66].

Monocytes migrate to the wound, differentiate into macrophages, phagocytose apoptotic cells and cellular debris, and trigger an inflammatory response [67]. Interestingly, CD4^+^ T cells were associated with wound healing, while CD8^+^ T cells negatively affected this process [68]. M1 macrophages have pro-inflammatory properties and express inflammatory cytokines and chemokines, including TNF-α, IL-1β, and IL-12 [69, 70]. M2 macrophages secrete anti-inflammatory factors such as IL-10/TGF-β. Transformation from M1 to M2 macrophages contributes to the transition from the inflammatory phase to the proliferative phase [71, 72]. During abnormal wound healing, large numbers of macrophages inappropriately release cytokines between the late inflammatory and proliferative phases, promoting pathologic scarring.

### Proliferation

The proliferative phase begins 3–4 days after injury and lasts 2–4 weeks. During the proliferative phase, a large number of new blood vessels and connective tissue are created, and the epithelium is re-formed [73, 74]. Reduces wound size through wound contraction and fibroproliferation. M2 macrophages promote tissue regeneration and ECM mass production by regulating the proliferation and migration of keratinocytes, fibroblasts, and endothelial cells [18, 75]. Fibroblasts begin to secrete large amounts of immature type III collagen into the matrix. As the tissue matures, neovascularization degenerates, and the ECM is rebuilt, entering the final remodeling phase [76].

### Remodeling

Remodeling is the third stage of wound repair and is considered the most clinically important stage, beginning approximately 3 weeks after injury and lasting up to 2 years [77]. The cellular components that accumulate in the wound leave the site of injury and the vascularization gradually subsides, leaving a wound that heals without cellular collagen [78]. TGF-β regulatory factor stimulates fibroblasts to produce elastin and fibronectin, which ultimately form elastic fibers that give the skin a certain elasticity and participate in the restoration of the dermal structure [79]. Collagen overload from haphazard type III collagen to more effective and stronger type I collagen, increasing the strength of scar tissue [80]. Reaches plateau about 7 weeks after trauma [24, 81]. Subsequently, stimulated by growth factors, fibroblasts differentiate into Myo-Fb [82]. These cells are responsible for wound contraction and are characterized by the expression of α-SMA [83]. Myo-Fb contains bundles of microfilaments attached to extracellular fibronectin, and these microfilaments generate contractile forces around the ECM, causing the wound edges to contract by 0.75 mm per day, ultimately leading to wound closure [18]. It has been shown that the biological behavior of skin fibroblasts is influenced by skin tension during scar formation, showing stronger HTS changes at 10-15% stretch [83].

In physiologic wound healing, Myo-Fb undergoes apoptosis upon completion of the epithelialization process, which stops ECM deposition and wound contraction [84]. However, in HTS and keloids, there are features such as increased inflammatory response, overexpression of growth factors, increased activation and proliferation of fibroblasts, and massive neovascularization, which creates conditions for excessive collagen deposition in pathologic scars [10, 85]. The inability of fibroblasts to undergo apoptosis after the completion of epithelialization leads to cause granulation tissue contraction and secretion of a large number of dense, disordered collagen fibers, which leads to the formation of HTS [10]. In keloids, fibroblast proliferation is more pronounced and resistant to FAS-mediated apoptosis [86]. Therefore, inhibiting the inflammatory response, regulating the proliferation and activation of fibroblasts, and antagonizing the deposition of ECM and vascularization are the keys to treating pathological scarring [87].

### Role of adipose-derived stem cells and secretome in scarring

**Fig. 3:**
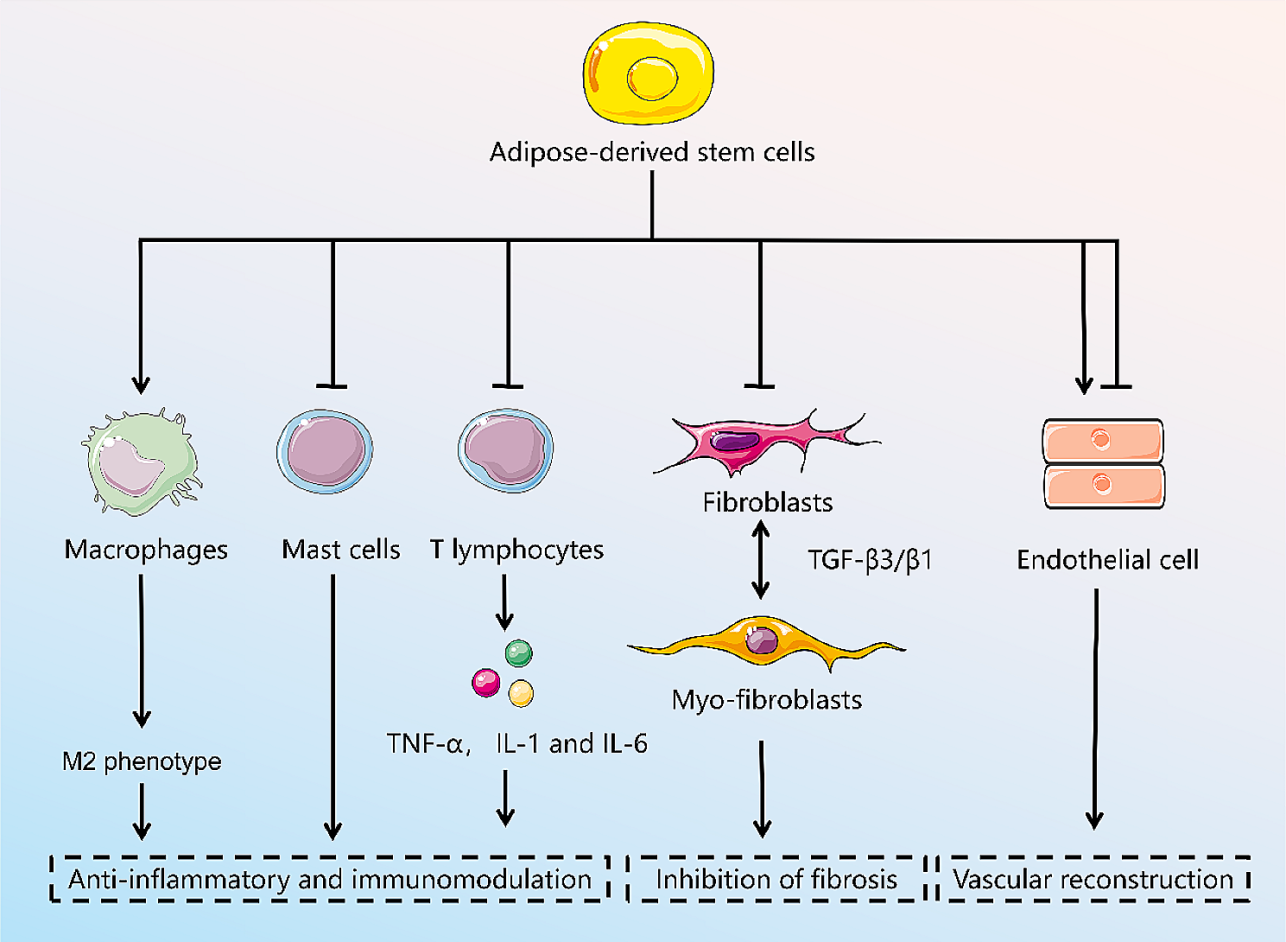
The mechanism of the treatment of adipose stem cells for hypertrophic scarring. Figure was drawn by the author

Based on the full understanding of the pathophysiology of HTS formation, ADSCs have come into the limelight for their anti-inflammatory, immunomodulatory, fibrosis inhibiting, and vascular reconstruction effects [88], as shown in Fig. 3. A large number of studies have shown that ADSCs can be stimulated by the traumatic inflammatory environment, initiating immune regulation and attenuating the inflammatory response [7, 89]. At the same time, they secrete a variety of cytokines, inhibit TGF-β1 and collagen expression, promote matrix metalloproteinase (MMP) expression, accelerate ECM decomposition, inhibit fibrosis, and effectively improve the appearance, texture, thickness, and softness of the scar [90]. Paracrine cytokines, exosomes, and other active substances have been reported to be major factors in the exertion of the biological effects of ADSC [49]. ADSC-CM and ADSC-Exo have recently gained attention as alternatives to conventional ADSC therapy [91]. The related in vivo, in vitro studies are summarized in Table 1. However, as the acquisition of ADSCs not only requires additional collagenase for digestion, but also in vitro amplification and culture, its safety and long-term survival rate need to be examined [92].

**Table 1:** Related animal and cell research with ADSCs

Year	Authors	Experimental models	Stem cell types	Injection methods	Stem cell source	Result
2023	Li et al. [54]	Mouse and hypertrophic scar fibroblasts (HSF)	Adipose derived stem cells (ADSCs) -culture medium (CM)	Subcutaneous injection	Subcutaneous adipose tissue	A novel peptide derived from ADSC-CM attenuated hypertrophic scar fibrosis in vitro and in vivo.
2022	Li et al. [111]	Nude mouse and HSFs	ADSCs	Injected in the hypertrophic scar implant	Bilateral thighs and buttocks	ADSCs efficiently cured hypertrophic scars by promoting the apoptosis of HSFs and by inhibiting their proliferation and migration.
2022	Zhou et al. [126]	Keloid fibroblasts (KFs)	ADSCs	—	Human subcutaneous adipose tissues	ADSCs strongly suppressed KFs’ proliferative and invasive behavior, negatively regulated KF apoptosis.
2022	Xie et al. [106]	Rabbit ear and HSFs	ADSCs	Intradermal injection	Groin adipose tissue	ADSCs demonstrated the ability to prevent hypertrophic scar (HTS) formation via inhibiting the proliferation and migration of the synthesis of extracellular matrix of HSFs.
2022	Li et al. [127]	KFs	ADSC- Exosomes (Exo)	—	Human subcutaneous adipose tissues	ADSC-Exos inhibited ECM deposition in keloids, which may have been mediated by inhibition of the TGF-β2/Smad3 and Notch-1 signaling pathways.
2021	Zhang et al. [121]	Rabbit ear and human KFs	ADSC-CM	—	Inguinal fat tissues of rabbits	ADSC-CM can downregulate the expression of α-SMA due to its anti-fibrosis effect and promote the rearrangement of collagen fibres, which is integral to scar precaution.
2021	Xie et al. [79]	HSFs and KFs	ADSCs	—	Human subcutaneous adipose tissues	ADSCs can affect the biological behavior of HSFs and KFs in vitro by regulating the TGF-β1/Smad pathway.
2021	Wu et al. [124]	KFs	ADSC-Exo	—	Liposuction	ADSCs-EXO may inhibit the expression of the TGF-β1/Smad pathway, and thereby inhibit the proliferation, migration, and collagen synthesis of KFs.
2021	An et al. [50]	Nude mouse	ADSC-secretome	Applied to wound	Human subcutaneous adipose tissues	ADSC secretome can be effectively produced via maturation process, and safely utilized to restore damaged tissue architecture in clinical cases.
2021	Li et al. [118]	Dorsal skin of mouse and HSFs	ADSC-Exo	Subcutaneous injection	Human subcutaneous adipose tissues	ADSC-Exo attenuated the deposition of collagen, the trans-differentiation of fibroblasts-to-myofibroblasts, and the formation of hypertrophic scar by in vitro and in vivo experiments.
2021	Xu et al. [72]	Dorsal skin of mouse	ADSCs	Intravenous injection	Dorsocervical subcutaneous region	During wound healing, ADSCs may have antifibrotic potential by altering macrophage polarization.
2021	Yuan et al. [51]	Dorsal skin of mouse and HSFs	ADSCs-Exo	Subcutaneous injection	ADSC	ADSC exosome therapy can decrease scar formation by inhibiting the TGF-β2/Smad3 signaling pathway.
2021	Zhou et al. [52]	Dorsal skin of mouse	ADSC-Exo	Subcutaneous injection	Human subcutaneous adipose tissues	ADSC can effectively promote skin wound healing while inhibiting scar formation at the wound.
2020	Lu et al. [56]	Dorsal skin of mouse, HSFs, and human microvascular endothelial cells (HMECs)	ADSC-extracellular vesicles (EV)	Subcutaneous injection	Abdomen subcutaneous adipose tissues	The miR-486‐5p secreted from ADSC‐EVs possesses the capacity to promote HSFs migration and proliferation as well as HMECs angiogenesis.
2020	Zhu et al. [57]	Rabbit ear	ADSC-EVs	Injected at the edge and base of wound	Abdomen subcutaneous adipose tissues	A local injection of hASC-EVs efficiently prevented hypertrophic scar formation by suppressing myofibroblast aggregation and collagen deposition.
2019	Luo et al. [135]	Dorsal skin of mouse	ADSCs	Injected into the surface of the wound	Inguinal subcutaneous adipose tissues	ADSC partially modulates microskin function and enhances wound healing by promoting angiogenesis in a full-thickness skin defect mouse model.
2018	Wang et al. [129]	KFs	ADSC-CM	—	Human subcutaneous adipose tissues	ADSC-CM downregulated the extracellular matrix-related gene expression of PAI-1, TIMP-1, and COL1.
2018	Chu et al. [113]	Scar fibroblasts	ADSCs	—	Inguinal subcutaneous adipose tissues	ADSCs can inhibit the mRNA and protein expressions of α-SMA and promote the mRNA and protein expressions of DCN in in vitro culture system.
2017	Foubert et al. [101]	Porcine	ADSCs	Nasal spray device	Inguinal fat pad	Autologous ADSC administration reduced the HTS development following deep-partial cutaneous injury.
2015	Zhang et al. [130]	Rabbit ear	ADSCs and ADSC-CM	Intralesional injection	Groin fat pads	ADSCs reduced the formation of rabbit ear hypertrophic scars by decreasing the α-SMA and collagen type Ι gene expression and ameliorating collagen deposition.

HSF, hypertrophic scar fibroblasts; ADSCs, adipose derived stem cells; ADSC-culture medium, ADSC-CM; KFs, keloid fibroblasts; HTS, hypertrophic scar; ADSC-Exo, ADSC-Exosomes; HMECs, human microvascular endothelial cells

### Direct differentiation

ADSC has a multi-differentiation potential and under certain conditions can differentiate into adipocytes, muscle cells, osteoblasts, chondrocytes, vascular endothelial cells, etc. ADSC have also been shown to be able to differentiate into keratin-forming cells [93, 94]. These results suggest that ADSC may also differentiate directly into epidermal and dermal cells to promote tissue regeneration and prevent scar formation in injured areas during wound healing [94, 95]. When seeded on a synthetic or naturally-derived scaffold in vitro, stem cells can be differentiated toward a desired phenotype by the appropriate composition, appropriate structure, and appropriate physicochemical and mechanical properties of the scaffold [96–99].

### Anti-inflammatory and immunomodulation

A moderate inflammatory response helps accelerate repair by removing inflammatory factors and cellular debris and fighting infection, whereas a chronic or excessive inflammatory response can lead to poor wound healing and pathologic scarring [100]. Therefore, surgeons emphasize modulating the inflammatory response in wounds to create an environment conducive to healing and reconstruction. It has been shown that ADSC upregulates inflammatory factors 2 weeks after injury (wound healing phase) and downregulates inflammatory factors 2 months after treatment (early scarring phase) [101].

After skin trauma, T cells, and macrophages persist, secreting and releasing a large number of inflammatory factors, especially IL-1, IL-6, and TNF-α, which aggravate the inflammatory response, leading to prolongation of the inflammatory phase and granulation proliferation to form scarring [102]. Xu et al. [103] found that ADSCs may greatly reduce scar formation during skin wound healing by modulating macrophage polarization. Liu et al. [104] found that ADSC-CM reduced the number of inflammatory cells in a keloid model and reduced angiogenesis and thus scar formation. In addition, insulin-like growth factor binding protein-7 secreted by ADSC inhibits the production of cytokines such as TGF-β1, VEGF, and IL-6 [105]. IL-10 is a potent anti-inflammatory cytokine, and in the context of pathologic scar formation, IL-10-modified ADSCs have been shown to have a beneficial effect on the proliferation, migration, and ECM synthesis of fibroblasts, accelerating wound healing time and decreasing scar area and scar prominence height [106]. In addition, ADSC-CM treatment promoted the M2 macrophage phenotype and induced the expression of IL-10 [107].

Activation of mast cells is a key factor in causing a chronic inflammatory response in scarring. Moderate amounts of mast cells contribute to wound healing; however, mast cells in HTS are 4 times larger than in normal skin, release inflammatory mediators histamine, IL-6, and IL-8, and promote inflammatory responses, inducing excessive ECM synthesis and vascularization in proliferative wounds, leading to HTS formation [108]. ADSC can further reduce proliferative scarring by inhibiting the number and activity of mast cells [94]. Prostaglandin E2 (PGE2) is produced with the help of the cyclooxygenase (COX)-1 or COX-2 and is the most abundant form of prostaglandin in the body [109]. It is well-known that PGE2 produced by ADSCs has an anti-inflammatory effect in the context of wound healing and pathologic scar formation, as it inhibits the proliferation and function of immune cells and induces macrophages to upregulate IL-10 expression. Yang et al. [110] using ADSC-CM cultured fibroblast model and in mouse keloid tissue found significantly increased levels of COX-2 and PGE2.

### Inhibition of fibrosis

Fibroblast activation and function are important for wound healing, but in pathological scarring, activation of fibroblasts themselves may lead to fibroblast deposition and scar formation [9, 111]. In pathological scarring, increased fibroblasts secrete TGF-β1, which reduces the dependence of fibroblasts on external growth stimulators and maintains a strong proliferative ability [112]. In addition, TGF-β1 accelerates fibroblast activation to Myo-Fb and secretes α-SMA causing scar contracture [83, 113]. Imai et al. [114] found that ADSC-CM was effective in inhibiting keloid contracture. TGF-β3, a key regulator of scarless healing, reduces early ECM deposition and resists scar formation by regulating the migration of keratinocytes and dermal fibroblasts [115]. The study showed that a high ratio of TGF-β3/β1 promotes scarless healing similar to fetal trauma [116, 117].

ADSC-Exos can stimulate the reconstruction of ECM by regulating fibroblast differentiation and gene expression, thereby promoting wound healing and preventing scarring [61]. Li et al. [118] showed that ADSC-Exos attenuated the expression of Col1, Col3, α-SMA, and p-Smad2/p-Smad3 in fibroblasts and attenuated collagen production. Wang et al. [119] found that intravenous injection of ADSC-Exos blocked the differentiation of fibroblasts to Myo-Fb and increased the ratio of TGF-β3 to TGF-β1, reducing the size of the scar in the wounds of mice. Li et al. [120] found that ADSC-CM reduced the expression of Col1, Col3, and α-SMA in vitro. HTS tissues cultured with ADSC-CM exhibited thinner, well-arranged collagen. Zhang et al. [121] optimized ADSC-CM for in situ cross-linking with polysaccharide hydrogels to significantly improve the therapeutic effect of inhibiting scar proliferation. Interestingly, recent studies have shown that adipocytes regenerate from Myo-Fb as a plastic cell type that can be used to treat human scars [122].

A characteristic manifestation of pathological scarring is excessive collagen and ECM deposition [123, 124]. Fibroblasts synthesize and remodel the ECM primarily by synthesizing MMPs and MMP inhibitors [125]. Excessive deposition of ECM can lead to scarring if it is not absorbed and remodeled in time [126, 127]. In HTS, the ratio between type I collagen and type III collagen (6:1) was lower than that in keloid (17:1), but the ratio in normal skin was 5:1. Reduced levels of MMP1 and MMP2 expression, and elevated levels of tissue inhibitor of metalloproteinase (TIMP)1, and TIMP2 expression are important mechanisms in the formation of HTS [128]. Wang et al. [129] showed that the expression of TIMP1 and the deposition of Col1 in keloid tissue were significantly reduced after co-culture of keloid tissue with ADSC-CM in vitro. In addition, the number of CD31 and CD34 vessels was significantly reduced. Thus, ADSC-CM exerted an anti-scarring effect by regulating collagen degradation alleviating the abnormal deposition of collagen, and inhibiting keloid angiogenesis. Zhang et al. [130] injected either ADSCs or ADSCs-CM into rabbit ear lesions resulted in a more normal appearance of the scar, a more regular organization of collagen, and a decrease in the expression of α-SMA and type Ι collagen. In addition, HGF is an antifibrotic cytokine involved in scar control. In pathological scar tissues, HGF secreted by ADSCs inhibited TGF-β expression, decreased the Col1/Col3 ratio and TIMP1 levels, and upregulated MMP-1 expression [131].

### Vascular reconstruction

During the wound healing phase, the rate of angiogenesis is at its peak during the proliferation phase, and the number of blood vessels decreases during the remodeling phase, when the anti-vascular factors gradually rise and the pro-vascular factors gradually degrade; once this balance is broken, it will lead to more capillary generation and promote scarring. Therefore, if angiogenesis can be inhibited under certain conditions, scarring will be reduced to a certain extent. Studies have shown that ADSC injection reverses the abnormal vascularization pattern of scar tissue and remodels the microvascular structure [132, 133]. Li et al. [127] using an ex vivo tissue explant model, and found that ADSC-Exos significantly suppressed COL production and disrupted the microvessel structure. Foubert et al. [101] found that treatment with ADSC in pigs promoted more normal collagen organization, lengthened elastic fiber length, and reduced vascularity. However, a large number of previous studies have come to the opposite conclusion that promoting intra-incisional vascularization attenuates pathologic scar formation, so extensive experiments are still needed to confirm the relationship between vascularization and scar formation [134, 135].

### Clinical trials of ADSCs

The first clinical application of ADSC was published in 2004 for the treatment of cranial defects in children, with new bone formation and eventual healing of the defect after 3 months of follow-up [136]. Since then, cells or cell fractions of adipose tissue origin have been increasingly used in clinical practice, including nanofat, SVF, ADSCs, and stem cell secretome. The therapeutic potential of these cells has been widely explored in a variety of diseases, including COPD, diabetic ulcers, Crohn’s disease, and others [137]. Many recent clinical trials of ADSCs applied to scarring have been completed or are ongoing, and these studies have shown that ADSCs can significantly induce skin repair and improve scar appearance [138–142]. However, in the follow-up of Gal et al. [143], no significant benefit of autologous fat graft treatment was found. The related clinical trials are summarized in Table 2. How to ensure that they play an active role in the treatment of pathologic scarring is an issue that needs to be focused on in subsequent studies. Although ADSCs show great potential in wound healing, there are still some challenges such as safety issues, determination of the optimal application method, and evaluation of long-term efficacy.

**Table 2:** Related clinic research with ADSCs

Year	Authors	Location	Status	Procedure	Number of patients	Results
2023	Kwon et al. [139]	South Korea	Completed	Stromal vascular fraction (SVF)	20	The experimental side showed significant improvements compared to the control side.
2022	Dongen et al. [141]	Netherlands	Completed	SVF	40	Injection of tSVF resulted in improved wound healing and reduced scar formation at 6 months postoperation, without any noticeable advantageous effects seen at 12 months.
2022	Behrangi et al. [140]	Iran	Completed	SVF	7	The use of SVF in the treatment of patients with acne scars accelerates the improvement of volume, area and depth of the scar by increasing collagen content and the dermal thickness,
2021	Kemaloğlu et al. [142]	Turkey	Completed	The fat and nanofat-enriched fat grafts injection	45	In breast reduction patients, simultaneous fat and nanofat-enriched fat grafting appears to be a safe and promising strategy for scar management.
2019	Abou et al. [138]	Egypt	Completed	Adipose derived stem cells (ADSC)	10	One injection of ADSCs is as effective as three sessions of fractional carbon dioxide laser in the treatment of atrophic acne scars.
2017	Gal et al. [143]	United States	Completed	Autologous fat grafting	6	Single treatment with autologous fat grafts did not improve mature pediatric burn scars when compared to normal saline injections.

SVF, Stromal vascular fraction; ADSC, Adipose derived stem cells

### Safety

The first issue to be addressed in the application of ADSCs in the clinic is safety. The immunoreactivity of animal-derived products during ADSC culture is a concern. The direct use of tissue aspirated by liposuction may be safer and more effective because it avoids in vitro manipulations that may alter the biological function of ADSCs and circumvent regulatory issues. In addition, there are concerns that transplantation of ADSCs may promote cancer cell proliferation, immune rejection, and treatment resistance through similar mechanisms that promote tissue regeneration by secreting growth factors, VEGF, and ECM proteins. Although the discussion on the safety of ADSCs is ongoing, there is no doubt that large randomized and controlled clinical trials are essential to reach a final safety recommendation.

### Separation and extraction standards

The lack of uniform and effective isolation and extraction methods is also an important reason that hinders the translation of ADSCs to the clinic. ADSCs obtained from different species and anatomical regions exhibit different characteristics. Currently, studies comparing the efficacy of ADSCs extracted by various extraction methods have not yet appeared, and therefore no standardized method has been defined. The equipment, reagents, and extraction environments used by different scholars also vary, and yields vary widely. In addition, the automated equipment used to isolate ADSC is classified as a Class III medical device by the U.S. Food and Drug Administration (FDA) and cannot be approved for clinical applications. In addition, the presence of collagenase in injectable ADSC products makes it difficult to obtain approval from authorities such as the FDA. Therefore, to produce large quantities of clinical-grade ADSC at present, more extensive research is needed to establish uniform and effective separation and extraction standards that comply with the Dynamic Pharmaceutical Manufacturing Practice, which will make a significant contribution to the development of adipose tissue engineering.

### Evaluation of treatment effects

Another challenge faced by ADSC-based therapies in clinical translation is the uncertainty of their clinical efficacy. In in vitro models, our findings have focused on studies with 2D cell culture systems, and these extensions typically ignore key features such as cell-cell and cell-extracellular cell matrix interactions, and tissue architecture. Animal models have been used in preclinical studies to evaluate the efficacy and safety of treatments, but there is a lack of standard animal models of scarring, and the differences in the mechanisms of scar formation between pigs and humans due to the high economic cost of pigs, the loose skin of rodents that shrinks rapidly after trauma, and the skin of the ear-free ear, which contains a cartilaginous layer, are not ideal animal models for the study of scarring.

## Conclusions

The advantages of ADSCs include easy extraction, less invasive, easy to obtain in large quantities, non-immunogenic, and no ethical issues involved. It has shown promising anti-scarring therapeutic effects through direct differentiation, immunomodulation, anti-inflammatory, anti-fibrotic, and modulation of angiogenesis. ADSC-based therapies, which mainly consist of ADSC and SVF, have been widely studied and applied in clinical practice. Through paracrine mechanisms, such as ADSC-CM and ADSC-Exo, are becoming increasingly popular with fewer ethical and safety concerns. However, there is some discrepancy between basic research and clinical practice. To apply ADSCs in clinical practice for the treatment of scarring still needs to go for continuous improvement, such as determining the optimal extraction method, dosage, and duration of action of ADSCs on scarring to maximize their effective effects. A large number of experiments and data are still needed to elucidate that ADSCs are indeed effective in scar control. Future studies should focus on solving these problems and promoting the wide application of adipose-derived stem cell therapy in wound healing. In conclusion, the author believes that in the near future, with the continuous improvement of industry laws and regulations, we will eventually overcome the difficult problem of scarring and apply ADSC technology in more related fields.

### Electronic supplementary material

Below is the link to the electronic supplementary material.

Supplementary Material 1

## Data Availability

Not applicable.

## Abbreviations

ADSCs: Adipose-derived stem cells
ADSC-CM: ADSC-conditioned medium
ADSC-Exo: ADSC-exosome
α-SMA: α-smooth muscle actin
bFGF: Basic fibroblast growth factor
BMSC: Bone marrow mesenchymal stem cell
COX: Cyclooxygenase
ECM: Extracellular matrix
EGF: Epidermal growth factor
HGF: Hepatocyte growth factor
HIF-1α: Hypoxia inducible factor 1α
HTS: Hypertrophic scarring
IL: Interleukin
MMP: Matrix metalloproteinase
MSCs: Mesenchymal stem cells
Myo-Fb: Myofibroblasts
PDGF: Platelet-derived growth factor
PGE2: Prostaglandin E2
SVF: Stromal vascular fraction
TGF-β1: Transforming growth factor-β1
TIMP: Tissue inhibitor of metalloproteinase
TNF: Tumor necrosis factor
VEGF: Vascular endothelial growth factor
