# Supplementary material for: Insights into the role of adipose-derived stem cells and secretome: potential biology and clinical applications in hypertrophic scarring
Source: Stem Cell Res Ther. 2024 May 12;15:137. doi: 10.1186/s13287-024-03749-6 (PMC11089711; doi:10.1186/s13287-024-03749-6)
Supplement: Supplementary file 1 — Supplementary Material 1 [file 13287_2024_3749_MOESM1_ESM.docx]

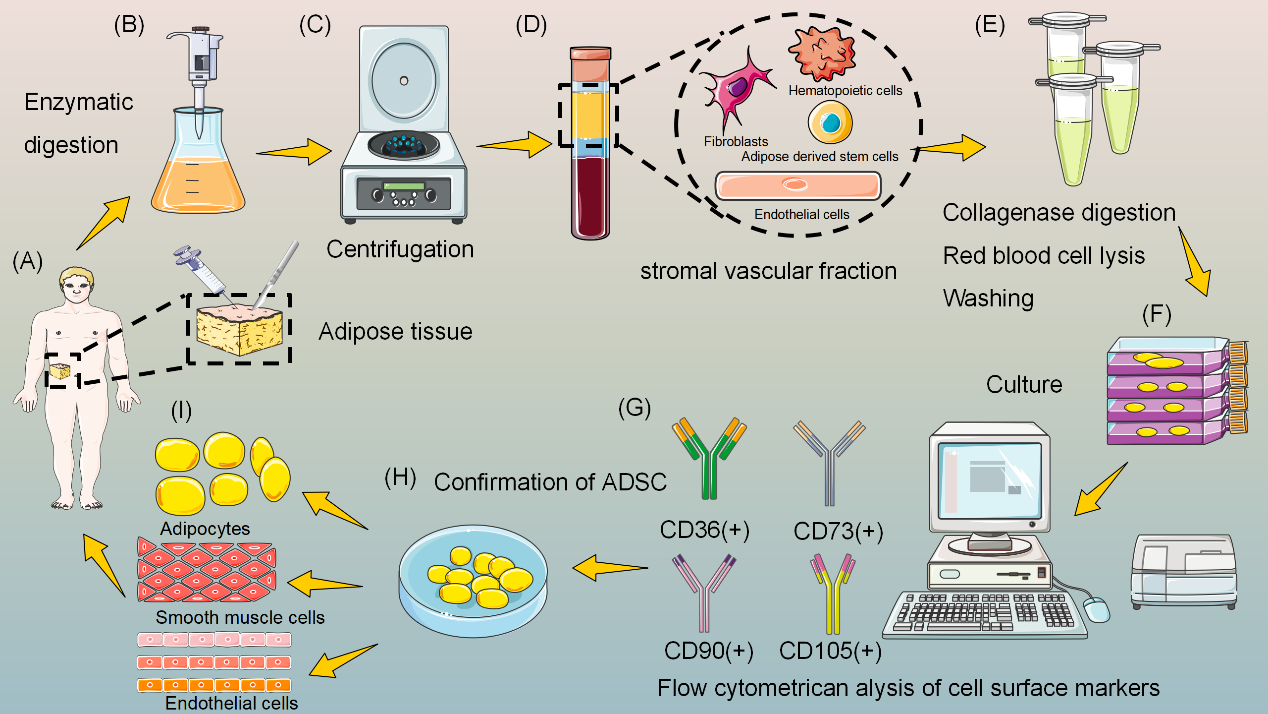


Fig. 1. The process of harvesting, isolation, and characterization of ADSC. Adipose tissue was obtained by liposuction or surgical excision, digested by enzymes, and centrifuged to isolate the stromal vascular fraction. After collagenase digestion, red blood cell lysis, and washing, the stromal vascular fraction was cultured and analyzed by flow cytometry for the presence of cell surface markers to confirm the presence of ADSC characteristics. After culture, they are applied to the human body. Figure was drawn by the author.


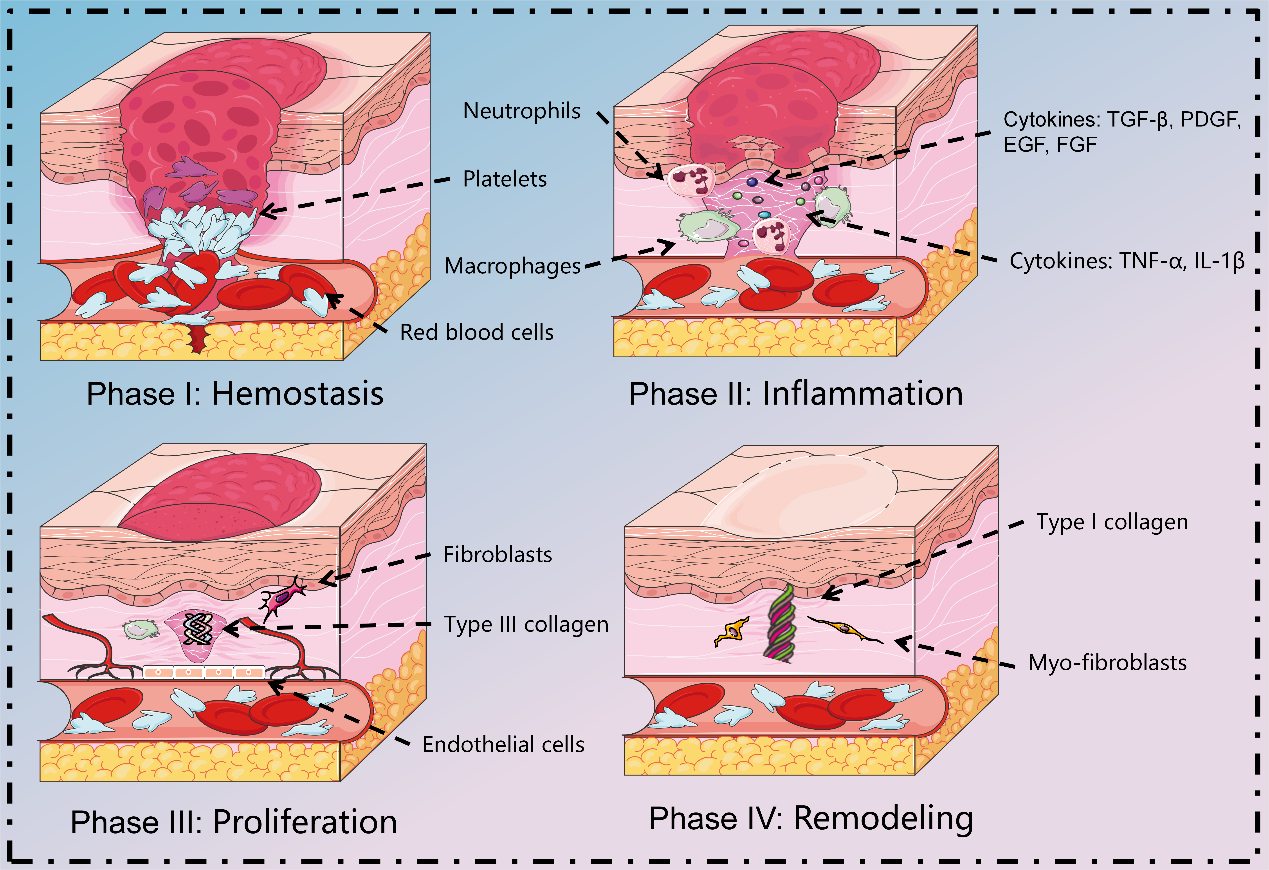


Fig.2 Normal healing process. Normal tissue repair involves many overlapping stages. After injury, hemostasis occurs initially through vasoconstriction and platelet aggregation. Subsequently, macrophages express inflammatory cytokines and chemokines, including tumor necrosis factor (TNF)-α, interleukin (IL)-1β, and IL-12, which recruit neutrophils and generate inflammation. Next, during the proliferative phase, macrophages promote tissue regeneration and ECM production by regulating the proliferation and migration of fibroblasts, and endothelial cells. As the tissue matures, neovascularization degenerates, and the ECM is rebuilt in the final remodeling phase. As granulation tissue is generated, fibroblasts differentiate into Myo-fibroblasts, which produce denser type I collagen and are responsible for wound contraction. Figure was drawn by the author.


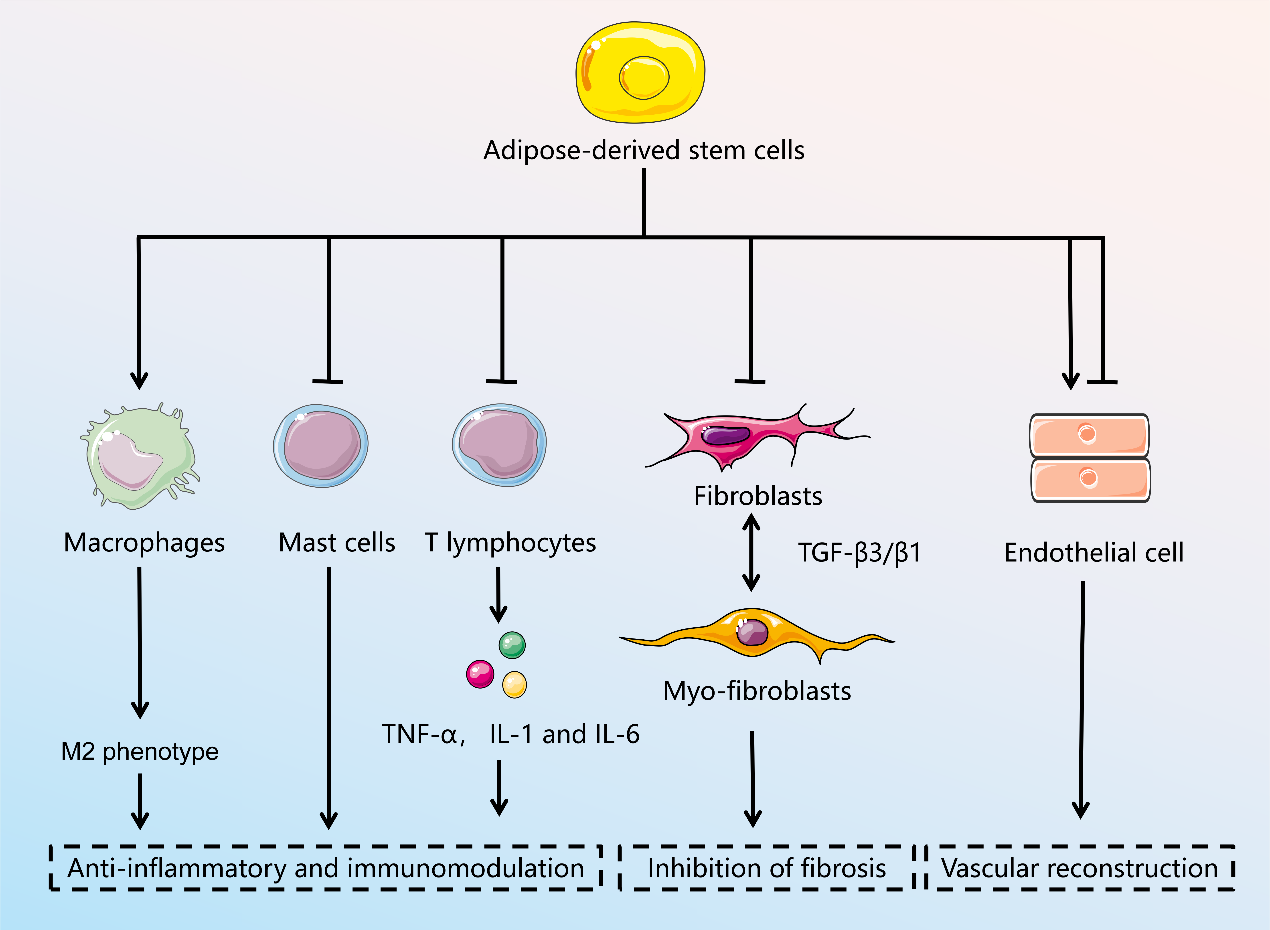


Fig.3 The mechanism of the treatment of adipose stem cells for hypertrophic scarring. Figure was drawn by the author.
